# MIP-2A Is a Novel Target of an Anilinoquinazoline Derivative for Inhibition of Tumour Cell Proliferation

**DOI:** 10.1371/journal.pone.0076774

**Published:** 2013-09-30

**Authors:** Mayuko Tokunaga, Hirokazu Shiheido, Noriko Tabata, Yuko Sakuma-Yonemura, Hideaki Takashima, Kenichi Horisawa, Nobuhide Doi, Hiroshi Yanagawa

**Affiliations:** Department of Biosciences and Informatics, Keio University, Yokohama, Japan; University of South Florida College of Medicine, United States of America

## Abstract

We recently identified a novel anilinoquinazoline derivative, Q15, as a potent apoptosis inducer in a panel of human cancer cell lines and determined that Q15 targets hCAP-G2, a subunit of condensin II complex, leading to abnormal cell division. However, whether the defect in normal cell division directly results in cell death remains unclear. Here, we used an mRNA display method on a microfluidic chip to search for other Q15-binding proteins. We identified an additional Q15-binding protein, MIP-2A (MBP-1 interacting protein-2A), which has been reported to interact with MBP-1, a repressor of the c-Myc promoter. Our results indicate that Q15 inhibits the interaction between MIP-2A and MBP-1 as well as the expression of c-Myc protein, thereby inducing cell death. This study suggests that the simultaneous targeting of hCAP-G2 and MIP-2A is a promising strategy for the development of antitumor drugs as a treatment for intractable tumours.

## Introduction

Globally, tumour formation is the leading cause of death in developed countries and the second most common cause of death in developing countries [1]. Despite improvements in the treatment of various common tumours, such as the combined use of chemotherapy and chemoradiation, colorectal tumours, lung tumours and multiple myeloma (a hematopoietic tumour) remain particularly intractable. Thus, the development of potent drugs is required to treat such intractable tumours.

**Figure 1:**
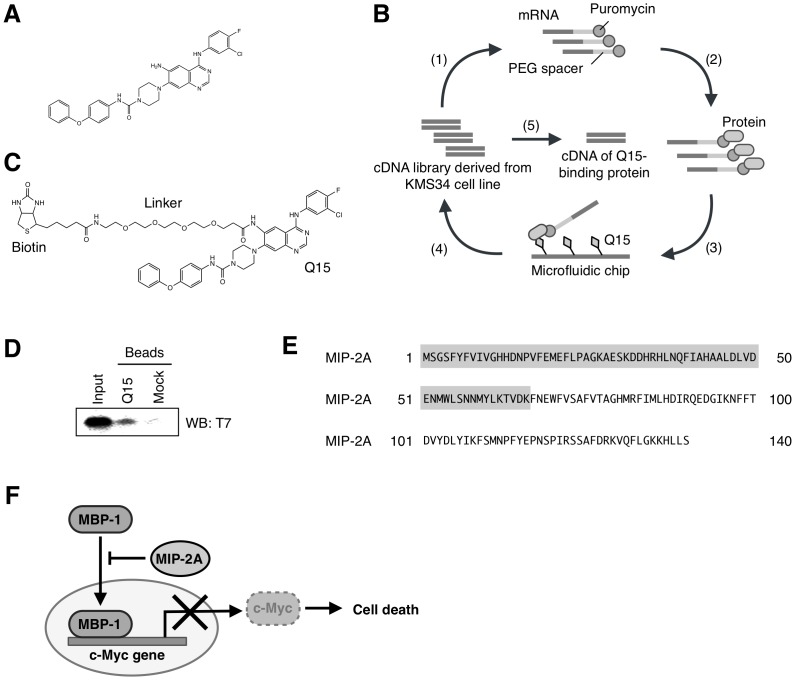
Schematic representation of the in vitro selection of Q15-binding protein by mRNA display. (A) Chemical structure of Q15. (B) Step 1: A cDNA library derived from KMS34 cells was transcribed and then ligated with a PEG-Puro spacer. Step 2: The resulting mRNA was in vitro translated to form a library of protein-mRNA conjugates. Step 3: The library was injected into a microfluidic chip on which Q15 was immobilised, and unbound molecules were washed away. Step 4: The bound molecules were eluted, and their mRNA portion was amplified by RT-PCR. The resulting DNA was used for the next round of selection and analysed by cloning and sequencing. (C) Chemical structure of biotinylated Q15. (D) The selected T7-MIP-2A_1-66_ was generated by *in vitro* translation and used in a pull-down assay with biotinylaed Q15 immobilized on streptavidin beads. Each fraction was separated by gel electrophoresis using 4–12% Bis-Tris Gel, followed by western blot analysis using an antibody against T7 tag. (E) The amino acid sequence of MIP-2A, identified as a Q15-binding protein by mRNA display. The grey-coloured sequence indicates the region (1–66 amino acids) identified by mRNA display selection. (F) MIP-2A binds to MBP-1, a transcriptional repressor of c-Myc. The nuclear transition of MBP-1 is inhibited by MIP-2A, resulting in aberrant expression of c-Myc and leading to suppression of cell death.

We recently used compound screening to identify an anilinoquinazoline derivative, Q15 (Fig. 1A), as a novel compound showing potent antitumor activity [2]. Additionally, using an mRNA display method [3]–[5], we identified hCAP-G2, a subunit of condensin II as a Q15-binding protein and confirmed that Q15 binds to the condensin II complex; accordingly, hCAP-G2 may affect chromosomal segregation in mitosis, leading to abnormal cell division and cell death [2]. However, it remains controversial whether only abnormal cell division caused by inhibition of condensin II leads to cell death.

In this present study, we aimed to search for additional targets of Q15 by using an mRNA display method in a microfluidic system, which is highly efficient for the selection of drug-binding proteins [6]. The use of the microfluidic chip also reduces the false-negative rate due to its low background. This feature made it possible to find another Q15-binding protein that was not detected in the previous system.

Consequently, we identified MIP-2A (MBP-1 interacting protein-2A) as a Q15-binding partner. MIP-2A has been identified as a MBP-1 binding protein using a yeast two-hybrid system. Prior to this, MBP-1 had been reported as a transcriptional repressor of c-Myc, binding to a TATA-box of the c-myc P2 promoter. Overexpression of exogenous MBP-1 leads to reduced c-Myc expression and cell death [7]. As c-Myc is a proto-oncogene product that plays a major role in the control of cell proliferation, MBP-1 exerts a regulatory effect on cell growth through regulation of c-Myc expression. Interaction of MIP-2A with MBP-1 inhibits the c-Myc repressor activity of MBP-1 [8]. We further confirmed that Q15 inhibits the interaction between MIP-2A and MBP-1 and thereby induces cell death by repressing the expression of c-Myc. Q15 may induce cell death by targeting both condensin II and MIP-2A.

## Results

### *In vitro* selection of Q15-binding proteins by mRNA display

We performed an mRNA display experiment to identify Q15-binding proteins (Fig. 1B). We first prepared a cDNA library derived from human multiple myeloma KMS34 cells, which are highly sensitive to Q15. From the cDNA library, we prepared mRNA-protein conjugates followed by affinity selection on biotinylated Q15 (Fig. 1C) immobilised on a microfluidic chip. After four rounds of selection, we analysed 18 clones. Among the candidates, we found that only MIP-2A_1–66_ bound to Q15 (Fig. 1D), while the other candidates did not. Also, we focused on the protein MIP-2A because it has been reported to be involved in apoptosis. Furthermore, the putative Q15-binding region MIP-2A_1–66_ (Fig. 1E), determined using the mRNA display method, is essential for interacting with myc-binding protein 1 (MBP-1) [7]–[12], which represses the transcription of c-Myc (Fig. 1F). Therefore, we hypothesised that Q15 inhibits the interaction between MIP-2A and MBP-1, thereby down-regulating the expression of c-Myc and leading to tumor cell death.

### Q15 inhibits the interaction between MIP-2A and MBP-1

**Figure 2:**
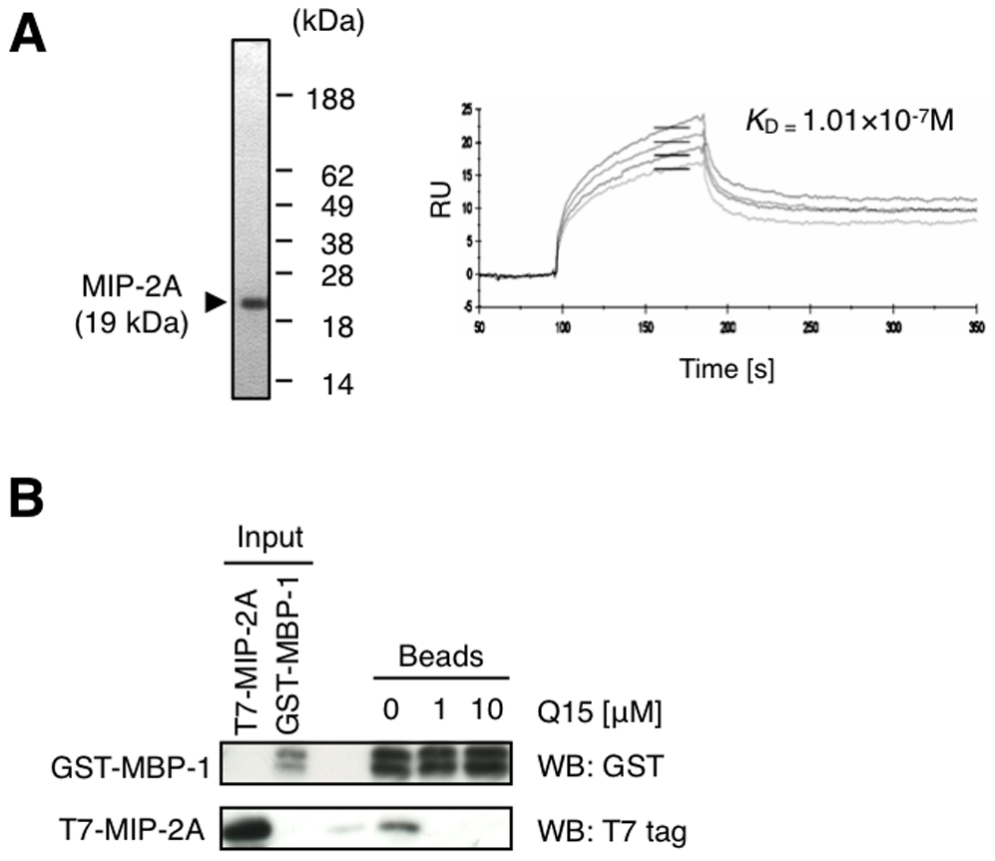
Q15 directly binds to MIP-2A and inhibits the interaction between MIP-2A and MBP-1. (A) Recombinant MIP-2A was expressed in *E. coli* and fractionated by gel-filtration chromatography. The fraction was then subjected to 15% SDS-PAGE followed by CBB staining (left). A representative biosensorgram of MIP-2A binding on SA sensor chips with immobilised biotinylated-Q15 is shown. The *K*_D_ value was determined (right). (B) T7-MIP-2A and GST-MBP-1 were generated by *in vitro* translation and used in a pull-down assay with glutathione sepharose in the presence of 0–10 µM free Q15. Each fraction was separated by 15% SDS-PAGE and analysed by western blotting with an antibody against T7 tag or GST.

To verify whether MIP-2A binds directly to Q15, we performed a kinetic analysis to evaluate the interaction between MIP-2A and Q15. We prepared MIP-2A recombinant protein in an *E. coli* expression system. The soluble fraction was treated with nickel affinity resin and then purified by gel-filtration chromatography on a Superdex 75 column (Fig. 2A, left). We then performed surface plasmon resonance analysis in which biotinylated Q15 was immobilised on an SA sensor chip. As a result, we found that MIP-2A binds to Q15 with a *K*_D_ value of 3.8×10^−7^ M (Fig. 2A, right).

We subsequently examined whether Q15 inhibits the interaction between MIP-2A and MBP-1, an interactor of MIP-2A as described above. We performed an *in vitro* assay of T7-MIP-2A binding to GST-MBP-1 immobilised on glutathione sepharose beads in the presence of 0-10 µM free Q15. Western blot analysis of each bead fraction showed that 1 µM Q15 was sufficient to disrupt the complex, and T7-MIP-2A was bound to GST-MBP-1 in the absence of Q15 (Fig. 2B). This result indicates that Q15 can inhibit the interaction between MIP-2A and MBP-1 *in vitro*.

### Q15 increases the intranuclear localisation of MBP-1

**Figure 3:**
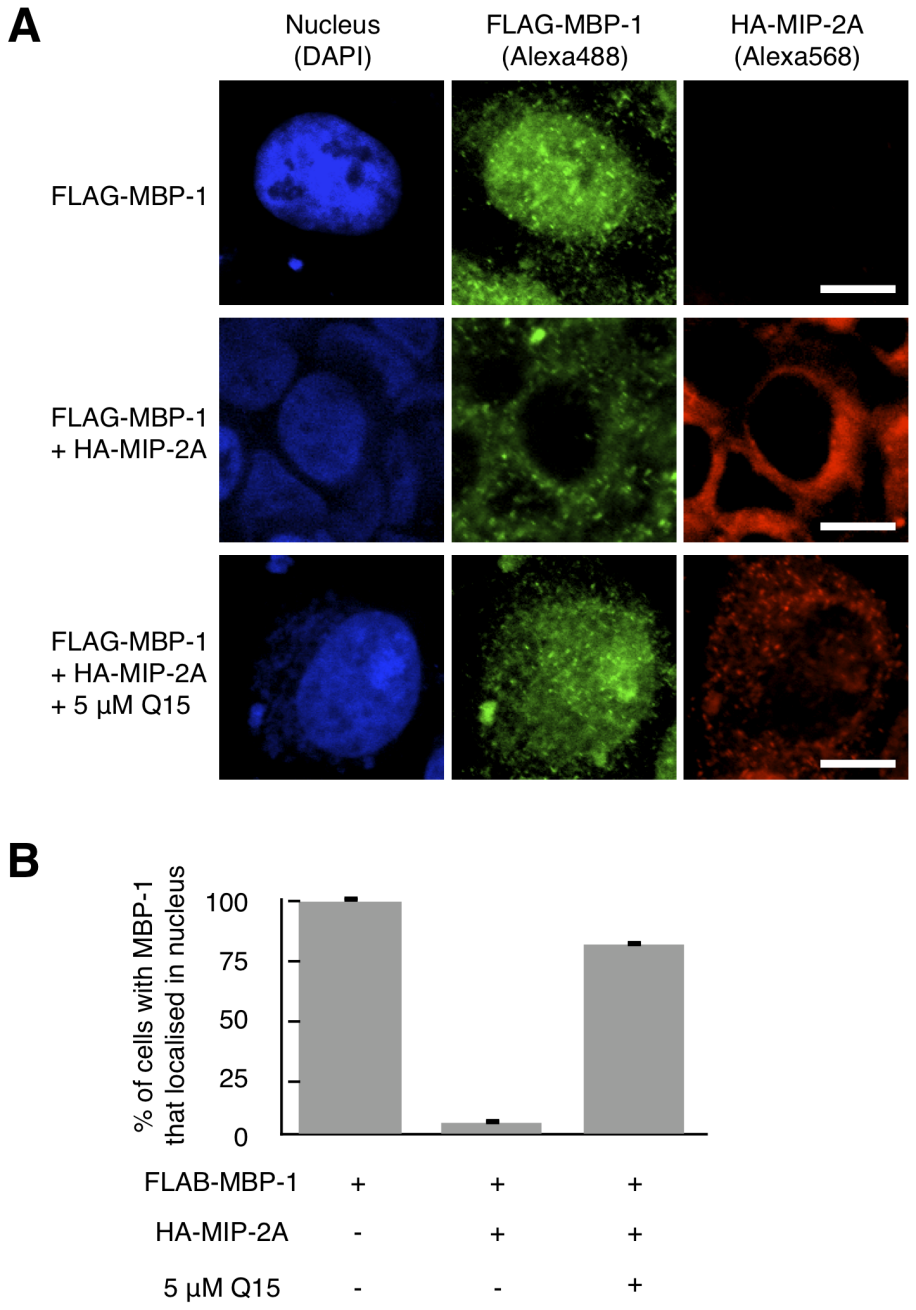
Q15 increases the intranuclear localisation of MBP-1. (A) HeLa cells were transfected with FLAG-MBP-1 with or without HA-MIP-2A. After 24 h, the cells were treated with 5 µM Q15 for an additional 24 h. Immunofluorescence staining with anti-FLAG (green) and anti-HA (red) was then performed. The samples were observed using confocal microscopy. Bar; 10 µm. (B) At least 50 cells per field were counted in each of three independent experiments. The ratio of cells with MBP-1 that localised in the nucleus was quantified.

MBP-1 mainly localises in nucleus and binds to the c-Myc P2 promoter to repress its transcription [9]. However, nuclear entry and the transcriptional activity of MBP-1 are prevented by MIP-2A [12]. Because Q15 inhibits the interaction between MIP-2A and MBP-1, as shown in Fig. 2B, the intranuclear localisation of MBP-1 should be increased by Q15. To evaluate this effect, we examined the cellular localisation of MBP-1 using immunofluorescence staining. HeLa cells expressing FLAG-MBP-1 with or without HA-MIP-2A were treated with 0 or 5 µM Q15 for 24 h followed by staining with anti-FLAG and anti-HA antibodies (Fig. 3A and B). When FLAG-MBP-1 was expressed alone, the majority of FLAG-MBP-1 localised in nucleus. However, when co-expressed with HA-MIP-2A, FLAG-MBP-1 mainly localised in the cytoplasm, thereby confirming that MIP-2A inhibits the intranuclear localisation of MBP-1. When the HeLa cells expressing both FLAG-MBP-1 and HA-MIP-2A were treated with Q15, intranuclear localisation of FLAG-MBP-1 was restored. Minor HA-MIP-2A staining in the nucleus after Q15 treatment may be due to non-specific binding. These results indicate that disruption of the MIP-2A-MBP-1 complex by Q15 increases the intranuclear localisation of MBP-1.

### Q15 down-regulates the expression of c-Myc

**Figure 4:**
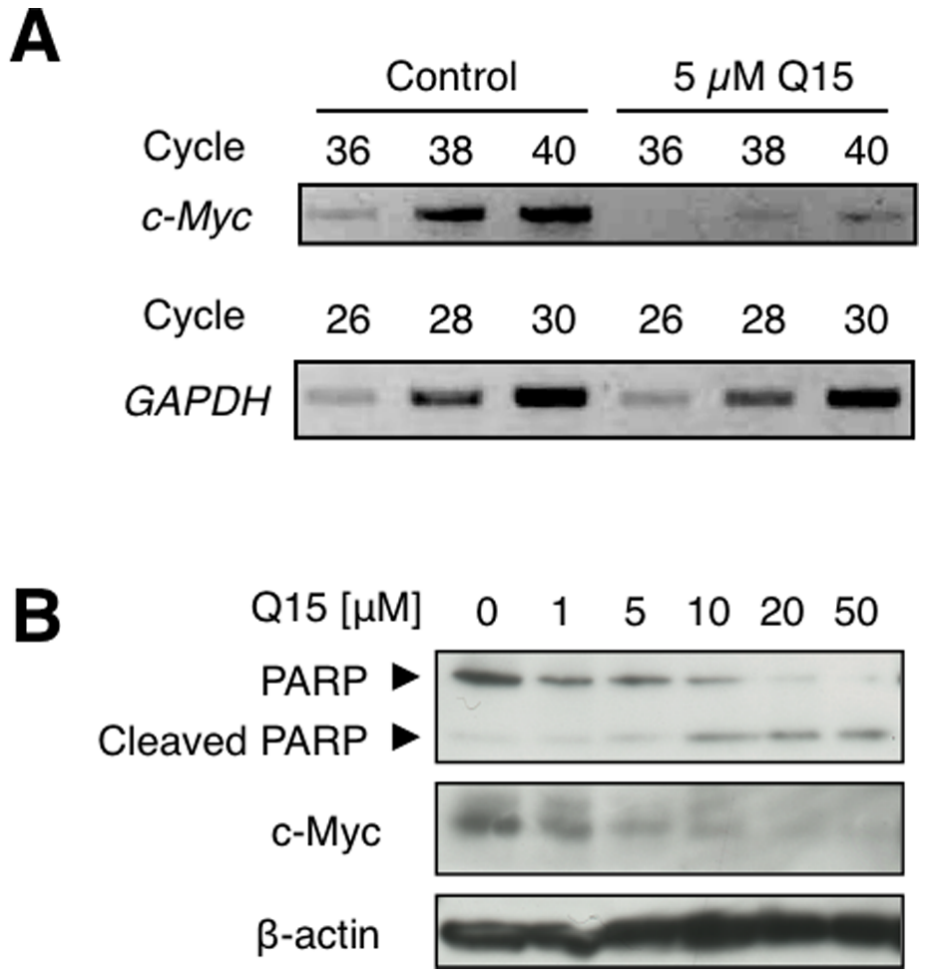
Q15 repressed the transcription of c-Myc. (A) HeLa cells were treated with 0 or 5 µM Q15 for 24 h. The total RNA was extracted from the cells and used as a template for RT-PCR using primers specific for *GAPDH* or *c-Myc*. (B) HeLa cells were treated with 0−50 µM Q15 for 24 h. The whole cell lysates were analysed by western blotting with an antibodies against PARP, c-Myc or β-actin.

MBP-1 is reported to be a repressor of c-Myc [9], and thus the enhanced intranuclear localisation of MBP-1 should lead to the down-regulation of c-Myc. To evaluate this effect, we examined whether Q15 affects the expression of c-Myc at the mRNA or protein level. We first analysed the mRNA level of c-Myc in HeLa cells treated with 0 or 5 µM Q15 for 24 h. RT-PCR analyses showed that when cells were treated with Q15, the c-Myc mRNA level decreased (Fig. 4A). To assess the expression level of c-Myc protein, HeLa cells were treated with 0−50 µM Q15 for 24 h followed by western blotting using anti-PARP or anti-c-Myc antibodies (Fig. 4B). As we have previously reported, the cleavage of PARP was detected by treatment with Q15 [2]. Furthermore, the c-Myc protein level also decreased in a Q15 concentration-dependent manner and correlated with the extent of PARP cleavage. These results suggest that the increase of the intranuclear localisation of MBP-1 caused by Q15 results in the down-regulation of c-Myc at the both mRNA and protein level.

### The inhibition of MIP-2A leads to cell death via the down-regulation of c-Myc

**Figure 5:**
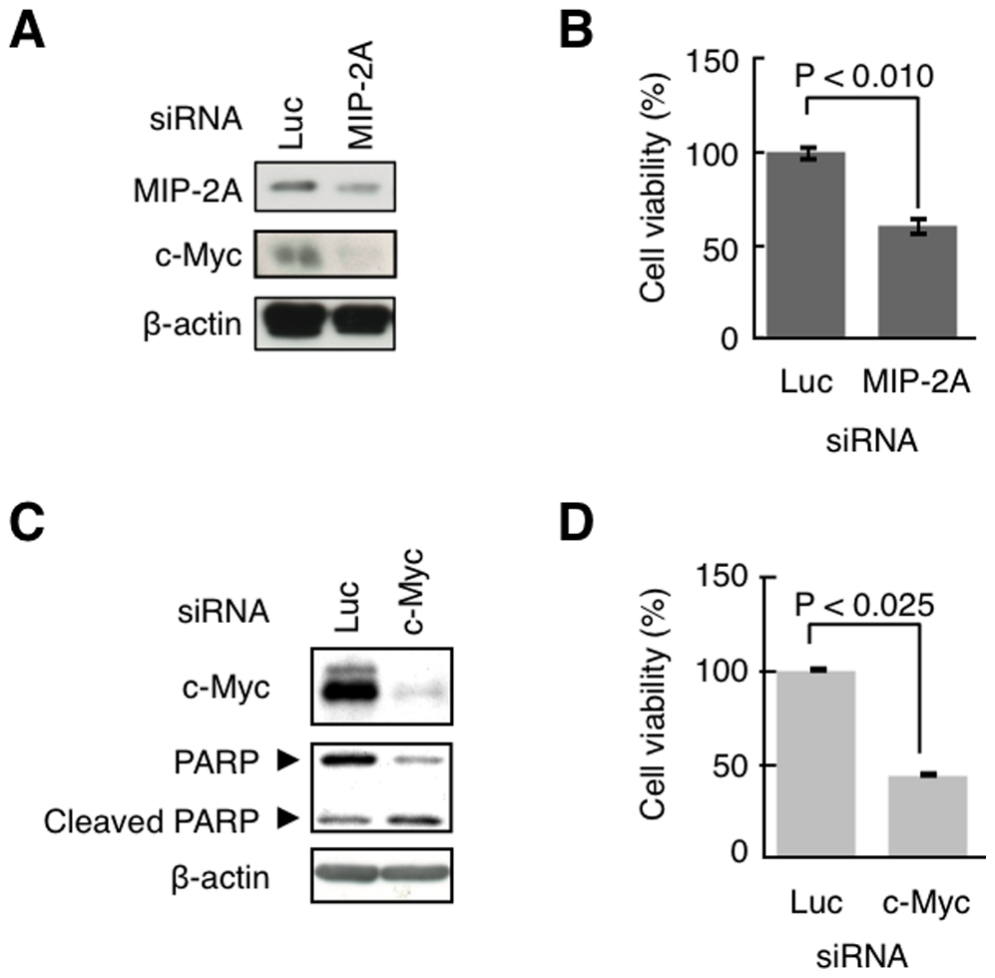
The knockdown of c-Myc or MIP-2A induces cells death. (A and C) HeLa cells were transfected with siRNA for luciferase (control), MIP-2A, or c-Myc. After 24 h, the whole cell lysates were analysed by western blotting with an antibody against c-Myc, PARP, MIP-2A, or β-actin. (B and D) Cell viability was determined using a WST-1 assay.

Finally, we examined whether the inhibition of MIP-2A specifically leads to the down-regulation of c-Myc and thereby results in cell death. Knockdown experiments were performed to test this hypothesis. We first examined the level of c-Myc protein expression after knockdown of MIP-2A. The results confirmed that the MIP-2A level was suppressed concomitantly with a decrease of the c-Myc protein level (Fig. 5A). Knockdown of MIP-2A also resulted in cell death (Fig. 5B). When c-Myc expression was suppressed with siRNA in HeLa cells, PARP was cleaved, as in the case of treatment with Q15 (Fig. 5C), suggesting that a decrease of c-Myc leads to apoptosis. Moreover, the cell viability decreased to less than 50% (Fig. 5D). These results suggest that inhibition of MIP-2A results in a decrease of c-Myc expression, leading to cell death.

## Discussion

In our recent study, Q15 was identified as a novel anilinoquinazoline derivative that represses the proliferation of several types of tumour cell lines, including intractable tumours [2]. Q15 showed a more potent activity than gefitinib [13], a well-established anilinoquinazoline derivative with a potent activity against non-small-cell lung tumours. Furthermore, we also reported that Q15 interacts with hCAP-G2, a subunit of the condensin II complex, and thereby induces a structural aberration of the chromosome in mitosis, leading to abnormal cell division [2]. However, whether such a defect in normal cell division is directly linked to apoptosis remains to be determined. For example, it is plausible that Q15 may target other proteins for the induction of apoptosis of tumour cells.

In this study, we performed a selection of Q15-binding proteins using an mRNA display method on a microfluidic chip. This approach has several advantages over selection on beads, including a lower non-specific binding and higher efficiency of enrichment [6], and was used to identify a drug-binding protein in a previous study [14]. Using this mRNA display selection system, we identified MIP-2A as an additional Q15-binding protein. Unexpectedly, hCAP-G2, which was identified in 6 of 100 clones in the previous selection, was not obtained, possibly because we analysed a smaller number of clones in this present study.

Several studies have reported that MIP-2A interacts with MBP-1, which acts as a repressor of the c-Myc promoter and thereby serves as an apoptosis inducer [8], [9]. We found that Q15 inhibits the interaction between MIP-2A and MBP-1, leading to a decrease in the c-Myc expression level. It has also been reported that depression of the proto-oncoprotein c-Myc by knockdown results in apoptosis [9]. However, in several cell lines, variations in the c-Myc protein levels do not affect the cell viability [15]. In our study, we confirmed that the knockdown of c-Myc in HeLa cells leads to apoptosis. Although cell viability decreased to below 50% after the knockdown of c-Myc, only a small percentage of PARP was cleaved. We think these results may indicate that inhibition of c-Myc leads to not only apoptosis but also lower cell proliferation. Moreover, cleavage of PARP was detected in the c-Myc knockdown trial, it was not detected in the case of knockdown of MIP-2A (data not shown), possibly because of insufficient repression of c-Myc due to the low knockdown efficiency of MIP-2A.

As shown in Figure S1, we found a negative correlation (correlation coefficient = −0.62) between expression level of c-Myc and sensitivity to Q15 in several cell lines we previously tested [2]. Five of the six Q15-sensitive cell lines (IC_50_ = 1.1−5.1), KMM1, KMS11, KMS34, RPMI8226, and SW480, expressed high levels of c-Myc, while HeLa cells had a low c-Myc expression level. KMS27 cells, which were less sensitive to Q15 (IC_50_ = 14.5) [2], expressed a low level of c-Myc. These results indicate that survival and proliferation of KMM1, KMS11, KMS34, RPMI8226, and SW480 cells may depend on c-Myc, i.e., the cells show “oncogene addiction” [16]–[19]. We speculate that Q15 may show potent activity against other cell lines that are addicted to c-Myc.

By selecting Q15-binding proteins using an mRNA display method, we identified MIP-2A_1–66_ as a putative Q15-binding region. Based on previous studies using X-ray crystallographic analysis [10], [11], we determined that MIP-2A_1–66_ corresponds to a part of the region responsible for interacting with other proteins including MBP-1. In addition to MBP-1, MIP-2A has also been reported to bind to and modulate the transcriptional activity of the orphan nuclear receptor SF-1 and homeodomain protein PITX1 [12]. Although Q15 may prevent the interaction of MIP-2A with these proteins as well as with MBP-1, the relationship between SF-1 and PITX1 and cell growth remains elusive.

As shown in Fig. 2B, double bands of GST-MBP-1 were detected after *in vitro* translation, suggesting that post-translational modification occurs. Previous findings by Sedoris *et al.* suggested that post-translational modification of MBP-1 affects its DNA-binding activity [20]. Although such modification, if indeed it occurs, may affect the interaction between MBP-1 and MIP-2A, further study will be needed to examine this issue.

In conclusion, we determined that Q15 binds not only to hCAP-G2 but also MIP-2A. Our data indicate that the inhibition of the interaction between MIP-2A and MBP-1 leads to a decrease in the expression of c-Myc protein, resulting in lower cell viability. Further analyses such as ChIP to examine directly whether binding of MBP-1 to c-myc P2 promoter is affected by Q15 would provide further support for our hypothesis. Either induction of abnormal cell division via the inhibition of hCAP-G2 or suppression of c-Myc via the inhibition of MIP-2A may be sufficient for the direct induction of cell death. Regardless of mechanism, Q15 clearly targets both hCAP-G2 and MIP-2A, resulting in the inhibition of tumour cell growth. Therefore, the simultaneous targeting of these proteins may be a promising strategy for the treatment of intractable tumours.

## Materials and Methods

### mRNA display selection for Q15-binding proteins

The affinity selection of target proteins of Q15 was performed as previously described [14] with modification of the bait drug.

### Preparation of plasmids

All primers used in this study are listed in Table 1. The cDNA of MIP-2A was amplified by PCR using T7-MIP2A-f and MIP2A-FLAG-His6-polyA-stop-r, BamHI-HA-MIP2A-f and MIP2A-HindIII-r, or NcoI-T7-MIP2A-f and MIP2A-FLAG-His6-Stop-XhoI-r from pOTB7-MIP-2A, and the PCR products were subcloned into the plasmid vector pCR3.3-TOPO, pcDNA3.1/Hygro(-) (Invitrogen, Carlsbad, CA, USA), or pET-15b (Novagen, Madison, WI, USA), respectively.

**Table 1:** Primers used in this study.

Primer	Sequence (5′ → 3′)
T7-MIP2A-f	ACAACAACAAACAACAACAAAATGGCTAGCATGACTGGTGGACAGCAAATGGCGAATTCCATGTCTGGGAGCTTCTACTTTG
MIP2A-FLAG-His6-polyA-stop-r	CTATTTTTTGTGGTGGTGGTGGTGGTGCTTGTCGTCATCGTCCTTGTAGTCGCTTAAAAGGTGTTTCTTCCC
BamHI-MBP1-f	GTGTGGGATCCATGATGATCGAGATGGATGGAACAG
MIP2A-HindIII-r	CACACAAGCTTCTAGCTTAAAAGGTGTTTCTTCCC
NcoI-T7-MIP2A-f	GTGTGTGTCCATGGCCGCTAGCATGACTGGTGGACAGCAAATGGCGAATTCCATGTCTGGGAGCTTCTACTTTG
MIP2A-FLAG-His6-Stop-XhoI-r	CACACACCTCGAGCTAGTGGTGGTGGTGGTGGTGCTTGTCGTCATCGTCCTTGTAGTCGCTTAAAAGGTGTTTCTTCCC
HA-MBP1-f	ACAACAACAAACAACAACAAAATGTATCCTTATGACGTGCCTGACTATGCCATGATCGAGATGGATGGAACAG
MBP1-FLAG-His6-polyA-stop-r	CTATTTTTTGTGGTGGTGGTGGTGGTGCTTGTCGTCATCGTCCTTGTAGTCCTTGGCCAAGGGGTTTCTG
BamHI-MBP1-f	GTGTGGGATCCATGATGATCGAGATGGATGGAACAG
MBP1-HindIII-r	CACACAAGCTTCTACTTGGCCAAGGGGTTTCTG
MBP1-f	ATGATCGAGATGGATGGAACAG
MBP1-FLAG-His6-polyA-stop-r	CTATTTTTTGTGGTGGTGGTGGTGGTGCTTGTCGTCATCGTCCTTGTAGTCCTTGGCCAAGGGGTTTCTG
O29-GST-f	ACAACAACAAACAACAACAAAATGTCCCCTATACTAGGTTATTGG
GST-MBP1-r	CTGTTCCATCCATCTCGATCATACGCGGAACCAGATCCG
MBP1-FLAG-His6-polyA-stop-r	CTATTTTTTGTGGTGGTGGTGGTGGTGCTTGTCGTCATCGTCCTTGTAGTCCTTGGCCAAGGGGTTTCTG
5′O29-f	GGAAGATCTATTTAGGTGACACTATAGAACAACAACAACAACAAACAACAACAAAATG
cMycRT2-f	TACCCTCTCAACGACAGCAG
cMycRT2-r	TCTTGACATTCTCCTCGGTG
GAPDH-f	CATGTTCGTGATGGGTGTGAACCA
GAPDH-r	AGTGATGGCATGGACTGTGGTCAT

The cDNA of MBP-1 was amplified from total RNA of KMS34 cells by RT-PCR using HA-MBP1-f and MBP1-FLAG-His6-polyA-stop-r or BamHI-MBP1-f and MBP1-HindIII-r, and the PCR products were cloned into plasmid vector pCR3.3-TOPO or pCMV-tag2A (Stratagene, Santa Clara, CA, USA), respectively. From the resulting plasmid, the MBP-1 coding DNA was amplified by PCR using MBP1-f and MBP1-FLAG-His6-polyA-stop-r. The PCR product was mixed with a GST-coding DNA fragment amplified by PCR using O29-GST-f and GST-MBP1-r. The mixture was used as a template for overlap-extension PCR and was subsequently subcloned into pCR3.3-TOPO.

### *In vitro* translation

For in vitro translation, the MIP-2A or MBP-1 coding DNA was amplified by PCR using 5′O29-f and MIP2A-FLAG-His6-polyA-stop-r or MBP1-FLAG-His6-polyA-stop-r from pCR3.3-MIP-2A or pCR3.3-MBP-1 plasmid, respectively. The PCR products were transcribed to RNA using SP6 RNA polymerase (Promega, Madison, WI, USA). The RNA was purified using an RNeasy mini kit (Qiagen, Hilden, Germany) and in vitro translated in the Wheat Germ Extract Plus system (Promega).

### Surface plasmon resonance analysis

The recombinant MIP-2A protein was prepared as follows. *Escherichia coli* strain BL21 (DE3) codon+ was transformed with pET15b-T7-MIP2A-FLAG-His×6. The cells were grown in LB at 37°C. When the OD_600_ reached 0.5–0.6, 1 mM IPTG was added, and the cells were incubated for an additional 6 h. The culture was centrifuged at 20,000 g, for 5 min at 4°C. The pellet was lysed using lysis buffer (50 mM Tris-HCl, pH 7.6, 200 mM NaCl) containing protease inhibitor cocktail (Sigma, St. Louis, MO, USA) and homogenised by sonication. The homogenate was centrifuged at 6,000 g for 20 min at 4°C, and the supernatant was collected as the soluble fraction. The soluble fraction was mixed with Cosmogel His-Accept (Nacalai Tesque, Kyoto, Japan) on a rotator for 2 h at 4°C. After removal of the supernatant, the beads were washed with lysis buffer containing 20 mM imidazole, and the protein was then eluted with 300 mM imidazole. The resulting eluates were separated by gel-filtration chromatography using a Superdex 75 column (GE Healthcare, Waukesha, WI, USA) with AKTA (GE Healthcare).

Binding kinetics were determined by surface plasmon resonance (SPR) analysis using a Biacore 3000 system (GE Healthcare). All experiments were performed at 25°C using TBS buffer (20 mM Tris-HCl, pH 7.5, 138 mM NaCl). Biotinylated Q15 was immobilised onto the SA sensor chip (GE Healthcare). The measurements were performed using 392.6 resonance units of the ligand and a flow rate of 20 µl/min. To determine the dissociation constants, four different concentrations of purified MIP-2A were injected. The injection period for association was 300 s. After each measurement, the chip surface was regenerated with 15 µl of Glycine 2.0 (GE Healthcare). The binding data were analysed with the steady-state affinity model using BIAevaluation software version 4.1 (GE Healthcare).

### Cell lines

The KMM1, KMS11, KMS27, RPMI8226, and KMS34 cell lines were a generous gift from Prof. T. Otsuki (Kawasaki Medical College, Kurashiki, Japan) [21] and were maintained in RPMI1640 medium with 10% foetal bovine serum and 1% penicillin/streptomycin. HEK293T (RIKEN Cell Bank, Ibaraki, Japan, 2002), HeLa (RIKEN Cell Bank, 2002), and SW480 (ATCC, 2005) cells were maintained in DMEM (Nacalai Tesque) with 10% foetal bovine serum, 1% penicillin and 1% streptomycin.

### Western blotting

HeLa cells were treated with 0–20 µM Q15 for 0–24 h. The cells were lysed with RIPA Buffer (50 mM Tris-HCl, pH 7.6, 150 mM NaCl, 1 mM EDTA, 0.5% sodium deoxycholate, 1% NP-40, 0.05% SDS) containing a protease inhibitor cocktail (Nacalai Tesque). Protein concentrations were determined using a BCA protein assay kit (Thermo, Rockford, IL, USA). Equivalent amounts of protein were separated by 8–15% SDS-PAGE followed by analyses with antibodies against Enolase (Santa Cruz, Santa Cruz, CA, USA), HA tag, c-Myc, caspase-9 (Cell Signaling Technology, Beverly, MA, USA), FLAG M2, β-actin, GST (Sigma), or MIP-2A (Abcam, Cambridge, MA, USA). The blots were developed using ECL chemiluminescence reagents (GE Healthcare), and the band intensity was quantified using ImageJ software (http://rsbweb.nih.gov/ij/).

### GST-affinity assay

T7-MIP2A-FLAG and GST-MBP1-FLAG proteins were prepared by *in vitro* translation. T7-MIP2A-FLAG was incubated with GST-MBP1-FLAG-immobilised Glutathione Sepharose 4B (Nacalai Tesque) in IPP150 buffer (10 mM Tris-HCl, pH 8.0, 150 mM NaCl, 0.1% NP-40) in the presence of 0–100 µM Q15 with rotation for 1 h at 4°C. The beads were washed three times with IPP150 and resuspended in SDS sample buffer (125 mM Tris-HCl, pH 6.8, 4% sodium dodecylsulphate, 10% sucrose, 0.01% bromophenol blue) containing 2% β-mercaptoethanol. The eluate was separated by 15% SDS-PAGE and analysed by western blotting.

### Immunofluorescence

HeLa cells on coverslips were transfected with pCMV-tag2A-MBP-1 and pcDNA3.1/Hygro(-) or pcDNA3.1/Hygro(-)-HA-MIP-2A using the Lipofectamine 2000 reagent (Invitrogen). After 24 h, the cells were treated with 5 µM Q15 for an additional 24 h. These cells were then fixed with 4% paraformaldehyde for 30 min followed by permeabilisation with 0.2% Triton X-100 (Nacalai Tesque) for 5 min. The samples were stained with antibodies against FLAG tag and HA tag followed by Alexa488-conjugated anti-mouse IgG (Invitrogen) and Alexa568-conjugated anti-rabbit IgG (Invitrogen). Photobleaching was prevented using Slow Fade Gold antifade reagent with DAPI (Invitrogen).

### Quantitative RT-PCR analysis

HeLa cells were treated with 20 µM Q15 for 24 h. Total RNA was then extracted from the cells and used as a template for RT-PCR reactions using a OneStep RT-PCR kit with primers cMycRT2-f and cMycRT2-r or GAPDH-f and GAPDH-r. For quantification, real-time RT-PCR was performed using a QuantiTect SYBR Green RT-PCR kit (Qiagen).

### WST-1 assay

HeLa cells were seeded in a 96-well plate and transfected with siRNA oligonucleotides against c-Myc, MIP-2A, or luciferase (Invitrogen) using Oligofectamine (Invitrogen). After 24−48 h, the number of viable cells was determined using the cell proliferation reagent WST-1 (Roche, Basel, Switzerland) according to the manufacturer's protocol.

## Supporting Information

Figure S1 **Expression level of c-Myc in several cell lines.** (A) Whole cell lysates of the indicated human tumor cell lines were analyzed by western blotting using antibody against c-Myc or β-actin. (B) The intensity of each band in (A) was quantified using ImageJ software. There was a negative correlation (correlation coefficient = −0.62) between c-Myc/β-actin ratio and IC_50_ value (see reference 2).
(PDF)
