# Supplementary figures and images for: MIP-2A Is a Novel Target of an Anilinoquinazoline Derivative for Inhibition of Tumour Cell Proliferation
Source: PLoS One. 2013 Sep 30;8(9):e76774. doi: 10.1371/journal.pone.0076774 (PMC3786957; doi:10.1371/journal.pone.0076774)

**A**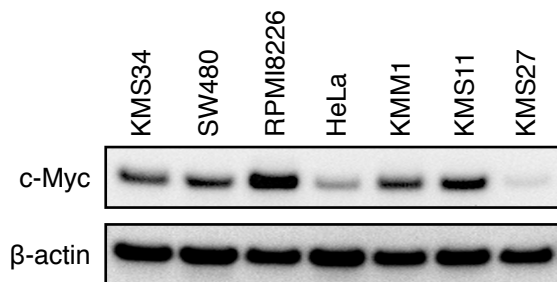**B**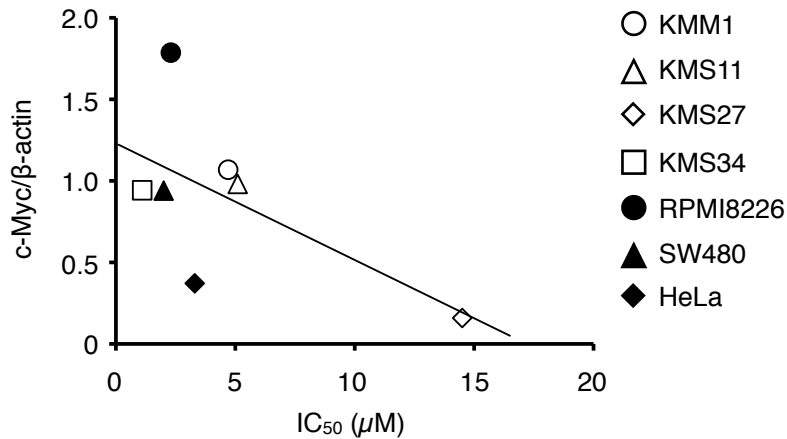

Supplement: Figure S1 — Expression level of c-Myc in several cell lines. (A) Whole cell lysates of the indicated human tumor cell lines were analyzed by western blotting using antibody against c-Myc or β-actin. (B) The intensity of each band in (A) was quantified using ImageJ software. There was a negative correlation (correlation coefficient = −0.62) between c-Myc/β-actin ratio and IC50 value (see reference 2). (PDF) [file pone.0076774.s001.pdf]
